# Acute and chronic mesenteric ischemia: single center analysis of open, endovascular, and hybrid surgery

**DOI:** 10.1186/s12893-022-01511-4

**Published:** 2022-02-13

**Authors:** Artur Rebelo, Marat Mammadov, Jumber Partsakhashvili, Carsten Sekulla, Ulrich Ronellenfitsch, Jörg Kleeff, Endres John, Jörg Ukkat

**Affiliations:** Department of Visceral, Vascular and Endocrine Surgery, Martin-Luther-University Halle-Wittenberg, Halle, Germany

**Keywords:** Mesenteric ischemia, Open surgery, Endovascular surgery, Hybrid surgery

## Abstract

**Background:**

The aim of the study was to analyse the outcome of open surgical, endovascular, and hybrid interventions in the treatment of acute (AMI) and chronic (CMI) mesenteric ischemia.

**Methods:**

Retrospective review of a cohort of mesenteric ischemia patients at a single tertiary referral center from 2015 to 2021. Primary end point was postoperative in-hospital mortality. Secondary end points were the number of bowel resections, duration of the procedure, length of postoperative intensive care treatment, length of hospital stay, revision surgery (number and type), and the nature and severity of postoperative complications according to Dindo-Clavien.

**Results:**

A total of 64 patients, 20 with CMI and 44 with AMI, underwent open, hybrid or endovascular surgery. Bowel resection was performed in 45.5% of the patients with AMI (29.5% small intestine, 2.3% colon and 13.6% both). There was no in-hospital mortality in the CMI cohort as compared to 29.5% in the AMI cohort (p = 0.03), with no differences regarding endovascular and open surgery (29.6 vs 29.4%). Severe postoperative morbidity (Dindo-Clavien ≥ 3) was also significantly more frequent in the AMI group when compared to the CMI group (20 vs 77.3%, p < 0.001). ASA classification and intensive care stay were identified as factors associated with mortality in AMI patients.

**Conclusions:**

Morbidity and in-hospital mortality are low in CMI patients, but substantial in AMI patients. Early diagnosis and open or endovascular treatment may be decisive for the outcome of these patients.

## Introduction

**Fig. 1:**
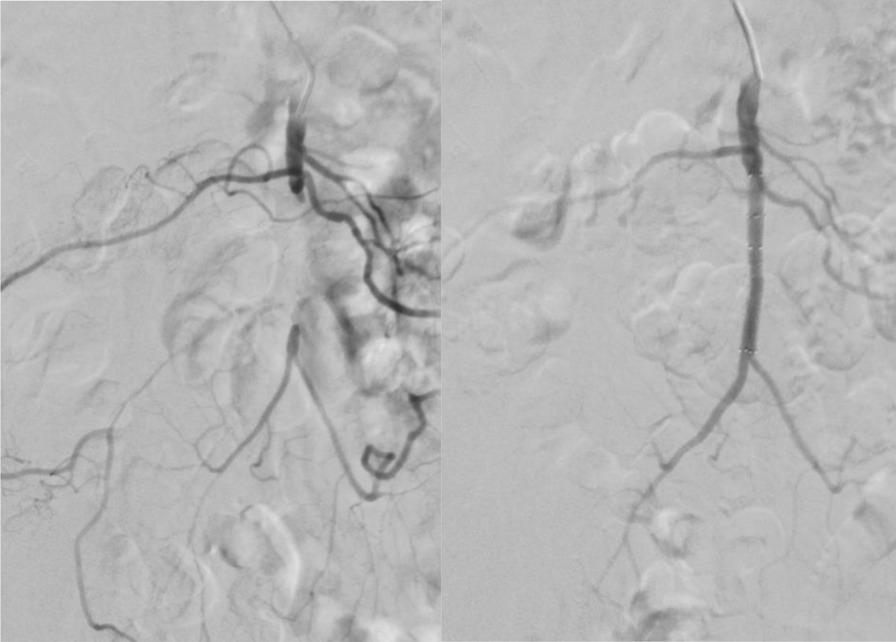
Patient with SMA occlusion treated with stent angioplasty

Mesenteric ischemia descriptions date back to 1900 [1, 2]. The first open atherectomy of the superior mesenteric artery was performed in 1958 [3]. Later in 1962, Crawford and DeBakey et al. described open revascularization of the celiac trunk and superior mesenteric artery [4]. Despite recent developments in endovascular and hybrid surgery, mesenteric ischemia mortality and morbidity rates are still high. (Fig. 1) [5].

Chronic mesenteric ischemia (CMI) is defined as symptomatic ischemia without irreversible tissue damage caused by insufficient blood supply to the gastrointestinal tract. The most common cause is atherosclerosis of the celiac trunk (CT), the superior mesenteric artery (SMA) or the inferior mesenteric artery (IMA) [6, 7]. CMI is the cause of abdominal pain in only 0.1% of hospital admissions for abdominal symptoms [8]. Symptoms are mostly postprandial abdominal pain (Stage II), "food anxiety", rest pain (Stage III) and weight loss. CMI remains an underdiagnosed disease [9]. Therefore, most patients present in the late stages of the disease with weight loss, chronic malnutrition, or intestinal infarction, which is then termed acute or acute on chronic mesenteric ischemia (AMI, Stage IV) [10, 11]. In addition, AMI can also be caused by arterial embolism and non-occlusive mesenteric ischemia [15]. The mortality of AMI is between 30 and 65% [12]. Bowel resection performed in an emergency setting is characterized by higher mortality [26]. CT angiography should be performed if AMI or CMI is suspected and is also the gold standard for follow-up after open and endovascular procedures [9, 13]. The use of CT scanning to diagnose mesenteric ischemia has increased over time [14]. Early diagnosis and intervention are critical to AMI.

Several guidelines were published on this matter [27, 28]. Endovascular and open surgery in asymptomatic patients with chronic mesenteric ischemia (CMI) is rarely indicated. On the other hand, symptomatic CMI should be treated to prevent acute mesenteric ischemia (AMI), bowel infarction, and death. It is still controversial which patients should undergo open or endovascular interventions [16].

The study aims to show the outcome of open surgical, endovascular and hybrid interventions in the treatment of AMI and CMI in a single tertiary referral centre.

## Methods

All patients 18 years and older at the time of surgery who underwent endovascular, open or hybrid surgery for mesenteric ischemia at the Department for Visceral, Vascular and Endocrine Surgery at the University Hospital Halle (Saale), Germany from 2015 to 2021 were included in the study. Patients with nonocclusive mesenteric ischemia and mesenteric venous occlusion were not included. Endovascular or hybrid treatment comprises mechanical thrombectomy, visceral artery angioplasty and stenting performed with or without laparotomy. Open revascularization comprises laparotomy with embolectomy, endarterectomy with or without patch angioplasty or bypass with prosthetic or venous grafting. Patients with AMI underwent emergency surgery. Patients with CMI underwent elective surgery. In our center we follow an endovascular first approach in the treatment of CMI. In AMI, when there is no clinical sign of bowel infarction, we perform an endovascular procedure. If there are clinical or radiological signs of bowel infarction, we perform a laparotomy and, depending on the extent of the arterial lesion, an exclusively open arterial bypass or hybrid procedure.

The primary outcome of the study is postoperative in-hospital mortality. Secondary outcomes are the number of bowel resections, type of operation (open surgical, endovascular, hybrid), duration of the procedure, length of postoperative intensive care treatment, length of hospital stay, and the nature and severity of postoperative complications according Dindo-Clavien Classification [21]. All outcomes and patients’ demographic characteristics and co-morbidities were collected by retrospective chart review. All data were anonymized prior to the analyses.

The study was approved by the ethics committee of the University Hospital Halle (Saale), Germany (ID 2021-031).

Pearson’s X^2^ test was used to identify independent factors associated with early death and postoperative morbidity. Mann–Whitney-Test was used for continuous and ordinal variables and Chi-square-test to the categorical variables. A P value of 0.05 determined statistical significance. IBM SPSS Statistics 27 was used to perform the analysis.

## Results

### Demographics and clinical characteristics

A total of 64 patients, 20 with CMI (elective surgery) and 44 with AMI (emergency surgery), underwent open, endovascular or hybrid surgery. In the CMI and AMI groups, 60% and 64.6% of patients were male, respectively. Mean age was 66.9 and 70.7 years in the CMI and AMI groups, respectively. Patients in the CMI group had higher prevalence of obesity and COPD and lower prevalence of diabetes mellitus, cardiac comorbidities, renal insufficiency, and history of malignancy. All patients were classified as ASA (American Society of Anesthesiology) score 3 or 4. Patients in the AMI group were classified as higher ASA risk when compared to the CMI group. A summary of relevant demographics and comorbidities are presented in Table 1. There were no statistically significant differences between both groups.

**Table 1:** Summary of the baseline and clinicopathologic features in 64 patients with AMI and CMI undergoing arterial revascularization from 2016–2021 (Mann–Whitney-Test for continuous and ordinal variables and Chi-square-test to the categorical variables used to compare CMI and AMI groups)

Variable	CMI (n = 20)			AMI (n = 44)			P value
	Open (n = 1)	Endovascular (n = 19)	Total	Open (n = 27)	Endovascular/Hybrid (n = 17)	Total	
Male Gender (%)	0%	63%	60%	48%	64.7%	54.4%	0.683
Age (years) Mean (SD)	75	66.5 (9.1)	66.9 (9)	67.9 (2.2)	75 (2.4)	70.7 (1.7)	0.16
ASA 3 (%)	100%	89.5%	90%	66.7%	76.4%	70.4%	0.087
DM (%)	0%	36.8%	35%	29.6%	47.1%	36.3%	0.916
Cardiac (%)	100%	73.7%	75%	81.5%	100%	88.6%	0.164
Renal Insufficiency (%)	0%	26.3%	25%	29.6%	52.9%	38.6%	0.287
Neoplasm (%)	0%	15.8%	15%	11.1%	17.6%	13.6%	0.884
Obesity (%)	0%	15.9%	15%	14.8%	0%	9%	0.483
COPD (%)	0%	26.3%	25%	14.8%	5.9%	11.3%	0.164

*AMI* acute mesenteric ischemia, *CMI* chronic mesenteric ischemia

*DM* diabetes mellitus

*COPD* chronic obstructive pulmonary disease

*ASA* American Society of Anesthesiologists Physical Status Classification System

### Etiology, classification, laboratory values and outcomes

In the AMI group, 27.3% had an embolic and 72.7% a thrombotic occlusion. Bowel resection was performed in 45.5% of the patients with AMI (29.5% small intestine, 2.3% colon and 13.6% both). Second-look laparotomy was performed in 27.3% of the patients. Regarding the CMI group, 25% of the patients were classified as stadium II and 75% as stadium III. The most often revascularized artery was the SMA in both groups. In the CMI group, all patients underwent revascularization. In the AMI group, 15.9% of the patients underwent bowel resection alone. In the CMI group, only one patient underwent open surgery while 19 patients received endovascular treatment. 80% of the patients received treatment for AMS and 20% for TC stenosis/occlusion. In the AMI group, of a total of 44 patients, 27 underwent open surgery and 17 endovascular treatments. The AMS was treated in 52.3% of the patients, TC in 13.6% and both in 23.5% of the patients.

Regarding the preoperative laboratory values, leukocytosis (gpt/l) and elevated lactate (mmol/l) were more frequent in the AMI group when compared to the CMI group (10.95 (± 1.69) vs 18.3 (± 1.9), p = 0.011 and 1.45 (± 0.27) vs 4.42 (± 0.79), p = 0.016). No statistically significant differences in CRP levels were observed between groups.

Concerning in-hospital mortality, no CMI patient died. In contrast, a mortality rate of 29.5% (p = 0.03) was observed in the AMI group, with no differences regarding endovascular and open surgery (29.6% vs 29.4% mortality). Severe morbidity (Dindo-Clavien ≥ 3) was also significantly more frequent in the AMI group when compared to the CMI group (77.3% vs 20%, p < 0.001). Endovascular surgery was associated with fewer postoperative complications when compared to open surgery (64.7% vs 85.2%, p < ***0.001***).

The length of intensive care stay, and hospital stay were different between the CMI and AMI groups (0.5 (± 0.45) vs 7.2 (± 1.9) days, p < 0.001 and 5.8 (± 1.2) vs 22.7 (± 3.3), p = 0.003). A summary of these results is presented in Table 2.

**Table 2:** Preoperative laboratory values, technical details, and postoperative outcomes in 64 patients with AMI and CMI undergoing arterial revascularization from 2016–2021 (Mann–Whitney-Test for continuous and ordinal variables and Chi-square-test to the categorical variables used to compare CMI and AMI groups)

Variable	CMI			AMI (n = 44)			P value
	Open (n = 1)	Endovascular (n = 19)	Total (n = 20)	Open (n = 27)	Endovascular/Hybrid (n = 17)	Total	
Etiology (%)	–	–	–	–	–	–	–
Embolic	–	–	–	33.3%	17.4%	27.3%	–
Acute on Chronic	–	–	–	66.7%	82.4%	72.7%	–
CMI Stadium (%)				–	–	–	–
2	0%	26.3%	25%	–	–	–	–
3	100%	73.7%	75%	–	–	–	–
Bowel Resection (%)	–	–	–	62.96%	17.65%	45.5%	–
Small intestine	–	–	–	40.7%	11.7%	29.5%	–
Colon	–	–	–	3.7%	0%	2.3%	–
Both	–	–	–	18.5%	5.88%	13.6%	–
Second-look laparotomy (%)	–	–	–	18.52%	41.18%	27.3%	–
Artery	–	–	–	–	–	–	–
None	0%	0%	0%	22.2%	5.9%	15.9%	–
AMS	100%	78.9%	80%	48.15%	58.8%	52.3%	–
TC	0%	21.1%	20%	14.8%	11.8%	13.6%	–
Both	0%	0%	0%	14.8%	23.5%	18.1%	–
Leukocytes(gpt/l)	18	10.6 (1.7)	10.95 (1.69)	20.14 (2.7)	15.4 (2.22)	18.3 (1.9)	0.011
CRP (mg/l)	30	37 (11.9)	37.5 (11.3)	96.12 (23.1)	110.97 (29.84)	101.89 (18.1)	0.072
Lactate (mmol/l)	2.2	1.4 (0.285)	1.45 (0.273)	4.267 (0.998)	4.665 (1.342)	4.42 (0.793)	0.016
In-Hospital mortality (%)	0%	0%	0%	29.6%	29.4%	29.5%	0.028
Dindo-Clavien ≥ 3	100%	15.8%	20%	85.2%	64.7%	77.3%	< 0.001
Surgery duration (min)	219	66.3 (8.1)	73.95 (10.8)	112.6 (14.1)	107.7 (24.16)	110.7 (12.6)	0.032
ITU Stay (d)	9	0.05 (0.229)	0.5 (0.45)	8.3 (2.8)	5.35 (1.87)	7.16 (1.85)	< 0.001
Hospital stay (d)	18	5.1 (1.01)	5.75 (1.16)	27.26 (4.7)	15.35 (3.68)	22.66 (3.3)	0.003

*AMI* acute mesenteric ischemia, *CMI* chronic mesenteric ischemia

*AMS* superior mesenteric artery

*CT* celiac trunk

*ITU* intensive care unit

### Factors associated with postoperative morbidity and mortality in AMI patients

The ASA classification was found to be associated with postoperative mortality in the AMI group. Patients who died had a longer intensive care stay (10 (± 12.9) vs 6 (± 12) days, p = 0.05), and more often bowel resections (61.5% vs 38.7%, p = 0.14) than those who survived.

Severe postoperative morbidity (Dindo-Clavien ≥ 3) was associated with bowel resections (55.8% vs 10%, p = 0.065) and inversely associated with second-look laparotomy rates (20.5% vs 50%, p = 0.066). A summary of these results is presented in Table 3.

**Table 3:** Pearson’s X2 test for factors associated with postoperative mortality and morbidity (Dindo-Clavien ≥ 3) in patients with acute mesenteric ischemia

Mortality	Yes (n = 13)	No (n = 31)	P value
Male gender (%)	46.2%	58.1%	0.469
Age (years)	72.8 (10.3)	69.77 (11.9)	0.832
ASA 3 (%)	23.1%	90.3%	< 0.001
DM (%)	53.8%	70.9%	0.118
Cardiac (%)	92.3%	87.1%	0.619
Renal failure (%)	46.1%	35.5%	0.507
Neoplasm (%)	0%	19.4%	0.088
Obesity (%)	7.7%	9.8%	0.834
COPD (%)	15.4%	9.68%	0.586
Etiology—Embolic (%)	15.39%	32.26%	0.252
Open surgical approach (%)	61.54%	61.29%	0.998
Bowel resection (%)	61.54%	38.71%	0.141
Second-look laparotomy (%)	30.78%	25.8%	0.736
Leukocytes (gpt/l) Mean (SD)	13.9 (7.8)	20.17 (13.64)	0.465
CRP (mg/l) Mean (SD)	89.49 (94.66)	107.1 (130.23)	0.603
Lactate (mmol/l) Mean (SD)	4.51 (3.33)	4.38 (5.935)	0.272
surgery duration (min) Mean (SD)	148.38(113)	94.4 (61.2)	0.482
ICU Stay (d)	10 (12.92)	5.97 (12)	0.05
Hospital stay (d)	13.85 (15.4)	26.35 (23.5)	0.144

Morbidity	Yes (n = 34)	No (n = 10)	P value
Male Gender (%)	60%	52.9%	0.694
Age (years)	72.1 (11.3)	65.8 (11.1)	0.283
ASA 3 (%)	67.6%	80%	0.452
DM (%)	38.2%	30%	0.634
Cardiac (%)	85%	100%	0.198
Renal Failure (%)	35.3%	50%	0.401
Neoplasm (%)	11.8%	20%	0.505
Obesity (%)	11.7%	0%	0.255
COPD (%)	11.8%	10%	0.877
Etiology—Embolic (%)	29.4%	20%	0.557
Open surgical approach (%)	67%	40%	0.114
Bowel resection (%)	55.8%	10%	0.065
Second-look laparotomy (%)	20.5%	50%	0.066
Leukocytes (gpt/l) Mean (SD)	19.98 (1.72)	12.66 (13.86)	0.601
CRP (mg/l) Mean (SD)	110.9 (124.8)	71.1 (101.56)	0.307
Lactate (mmol/l) Mean (SD)	4.64 (4.71)	3.66 (7.065)	0.293
surgery duration (min) Mean (SD)	125.2 (80.07)	62.7 (76.9)	0.36
ICU Stay (d) Mean (SD)	7.79 (13.298)	5 (8.138)	0.352
Hospital stay (d) Mean (SD)	26.6 (23.14)	9.2 (9.331)	0.494

*AMI* acute mesenteric ischemia, *CMI* chronic mesenteric ischemia

*ICU* intensive care unit

*DM* diabetes mellitus

*COPD* chronic obstructive pulmonary disease

*ASA* American Society of Anesthesiologists Physical Status Classification System

## Discussion

In this retrospective study, we report our single center experience regarding the treatment of CMI and AMI, both with endovascular and open surgery.

The major finding from this study concerns the zero in-hospital mortality in CMI patients and the elevated in-hospital mortality in the AMI group. Severe postoperative morbidity (Dindo-Clavien ≥ 3) was also significantly more frequent in the AMI group when compared to the CMI group (20% vs 77.3%, p < 0.001). Endovascular surgery had fewer postoperative complications in AMI patients when compared to open surgery (64.7% vs 85.2%), not affecting mortality rates (29.6% vs 29.4). An elevated leukocyte count and lactate levels were present in the AMI group when compared to the CMI group. Finally, ASA classification and longer intensive care stay were identified as factors associated with mortality in the AMI group.

Our results regarding outcomes of AMI are comparable with a 12-year retrospective analysis in which 72 patients with AMI were analyzed. Perioperative morbidity and 30-day mortality rates were 39% and 31%, respectively, and second-look surgery was performed in 53% of the patients [12]. In another retrospective study, data from a 20-year period revealed a 30-day mortality rate of 27% in the 1990s and 17% during the 2000s. As in our study, no significant differences in outcomes between open and endovascular revascularization were observed [17]. In another retrospective analysis, summarizing a 12-year experience with endovascular treatment of AMI due to embolic occlusion of the SMA﻿, the total in-hospital mortality was 27.0%. Laparotomy was performed in 73.0% and bowel resection in 40.5% of the patients [20]. In a meta-analysis of 30-day mortality after open and endovascular therapy of AMI, five non- randomized studies were included. Endovascular therapy had lower bowel resection rates (OR 0.37, p = 0.03) and lower 30-day mortality rates (OR 0.50; p = 0.002) when compared to open surgery. The pooled overall 30-day mortality rate after endovascular therapy was 17.2% compared with 38.5% after open surgery [6].

Concerning patients with CMI, we observed no mortality or severe (Clavien-Dindo ≥ 3) morbidity. These findings could be related to the small patient collective. Nevertheless, in another retrospective analysis, similarly low mortality rates were observed. In a retrospective study from the Mayo Clinic (Rochester, Minnesota, USA), 343 patients showed a procedure-related mortality of 2.6% [18]**.** Given these favorable outcomes, CMI should be treated timely and before disease progression exposing patients to acute or acute on chronic disease at a higher age, in a poorer physical status and with co-morbidities. Therefore, early diagnosis of CMI and presentation in a vascular surgery center for treatment already at Stage II may be decisive for a better outcome of these patients.

In our analysis, no significant difference in terms of mortality between endovascular and open treatment for AMI was observed, despite higher morbidity rates on the open surgery group. In an analysis of register data from the Johns Hopkins Hospital, Baltimore, USA, 679 patients underwent vascular intervention for AMI. Mortality was significantly higher after open revascularization compared with endovascular intervention (39.3% vs 24.9%; P = 0.01) [19]. A meta-analysis regarding mortality after open and endovascular revascularization for CMI was published within the ESVS guidelines. In single center cohorts from highly specialized centers, no difference in mortality was identified (OR 1.12). In administrative data from the Nationwide Inpatient Sample from the USA, the mortality was lower ﻿after endovascular compared to open revascularization (OR 0.20) [6].

We observed a higher leukocyte count and elevated lactate levels in the AMI group when compared to the CMI group. According to the recent ESVS guidelines, in patients with acute abdominal pain, D-dimer measurement is recommended to exclude AMI. In contrast, lactate measurement is not recommended to diagnose AMI [6]. In our study, no data on preoperative D-dimer was available, as it is not commonly used at our centre in this context.

In our analysis, ASA classification and length of intensive care stay were associated with mortality in patients with AMI. Some small single center studies showed comparable results [22–25]. In another retrospective study, congestive heart failure and chronic kidney disease predicted postoperative mortality, and bowel resection and cerebrovascular disease predicted postoperative morbidity [17].

Our study has some limitations. The main drawback is that it is based on a small number of patients. In addition, the retrospective design is another significant limitation, increasing the risk of bias considerably. Therefore, the results should be carefully interpreted, and applied. Nevertheless, the findings of this work may provide useful information for clinicians treating mesenteric ischemia and should be included in future meta-analyses.

## Conclusion

Mesenteric ischemia remains a challenge. Morbidity and in-hospital mortality are low when treating CMI and high for AMI. Early diagnosis and open or endovascular treatment may be decisive for the outcome of these patients. Which treatment is better for which indication remains an open question and should be addressed in future studies.

## Data Availability

The data that support the findings of this study are available from the authors, but restrictions apply to the availability of these data, which were used under license for the current study, and so are not publicly available. Data are however available from the authors upon reasonable request and with permission of ethics committee of the University Hospital Halle (Saale).
